# Supplementary material for: Gene Expression Dysregulation in Whole Blood of Patients with Clostridioides difficile Infection
Source: Int J Mol Sci. 2024 Nov 25;25(23):12653. doi: 10.3390/ijms252312653 (PMC11641058; doi:10.3390/ijms252312653)
Supplement: Supplementary file 1 [file ijms-25-12653-s001.zip › ijms-3246045-supplementary.pdf]

Gene expression dysregulation in whole blood of patients with *Clostridioides difficile* infection

Supplementary Material

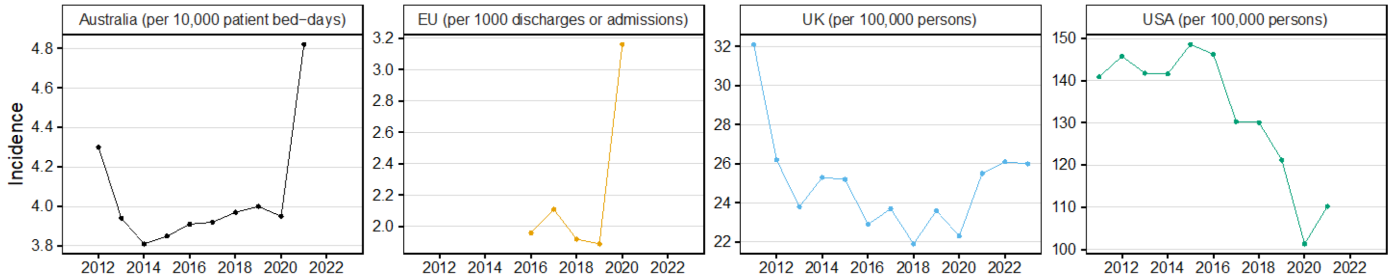

Figure S1: *C. difficile* incidence rate across countries between year 2012 and first quarter of 2023 at time of publication. The rate is based on total (healthcare and community acquired) cases in the UK and USA and hospital cases in EU and Australia. The EU report is based on different participation of countries/hospitals each year (crude incidence per 1,000 discharges or admissions). A two-step testing algorithm for *C. difficile* came into effect in April 2012 in the UK which reduced the number of false positive cases [2, 5, 6, 183].

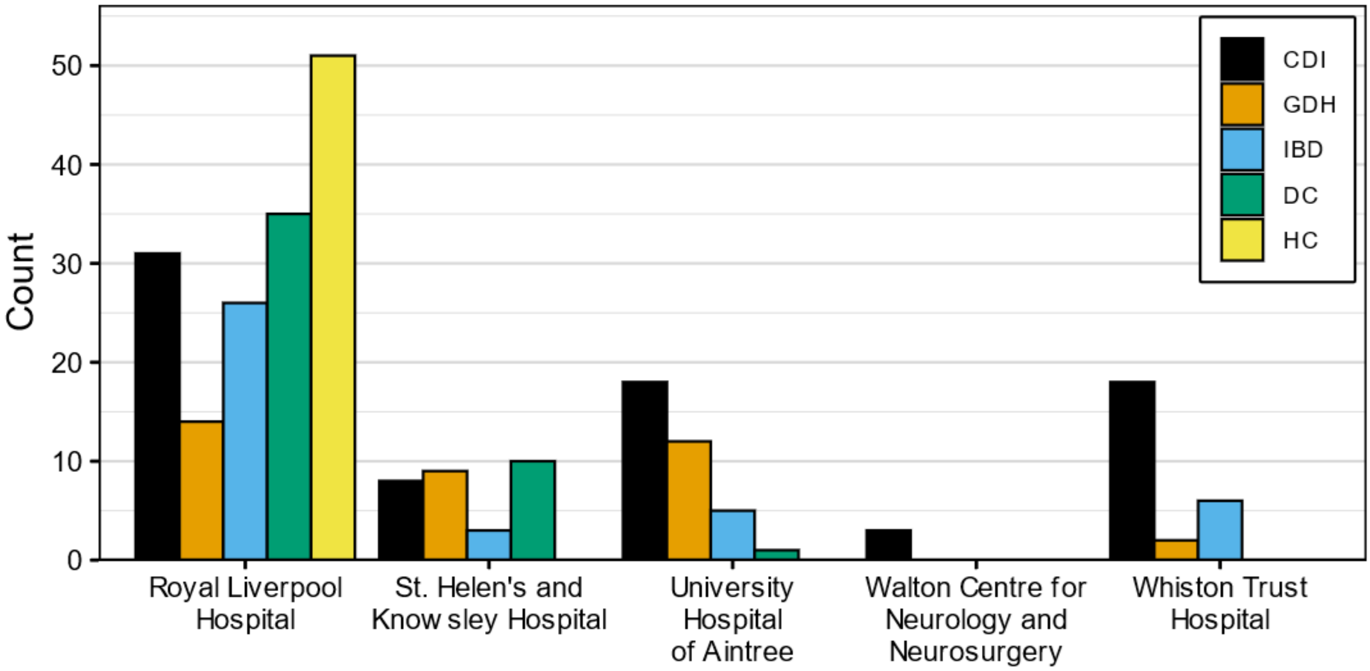

Figure S2: Distribution of condition among sites. *CDI: toxigenic C. difficile infection, GDH: non-toxigenic C. difficile infection, IBD: inflammatory bowel disease, DC: diarrhoea controls, HC: healthy controls.*

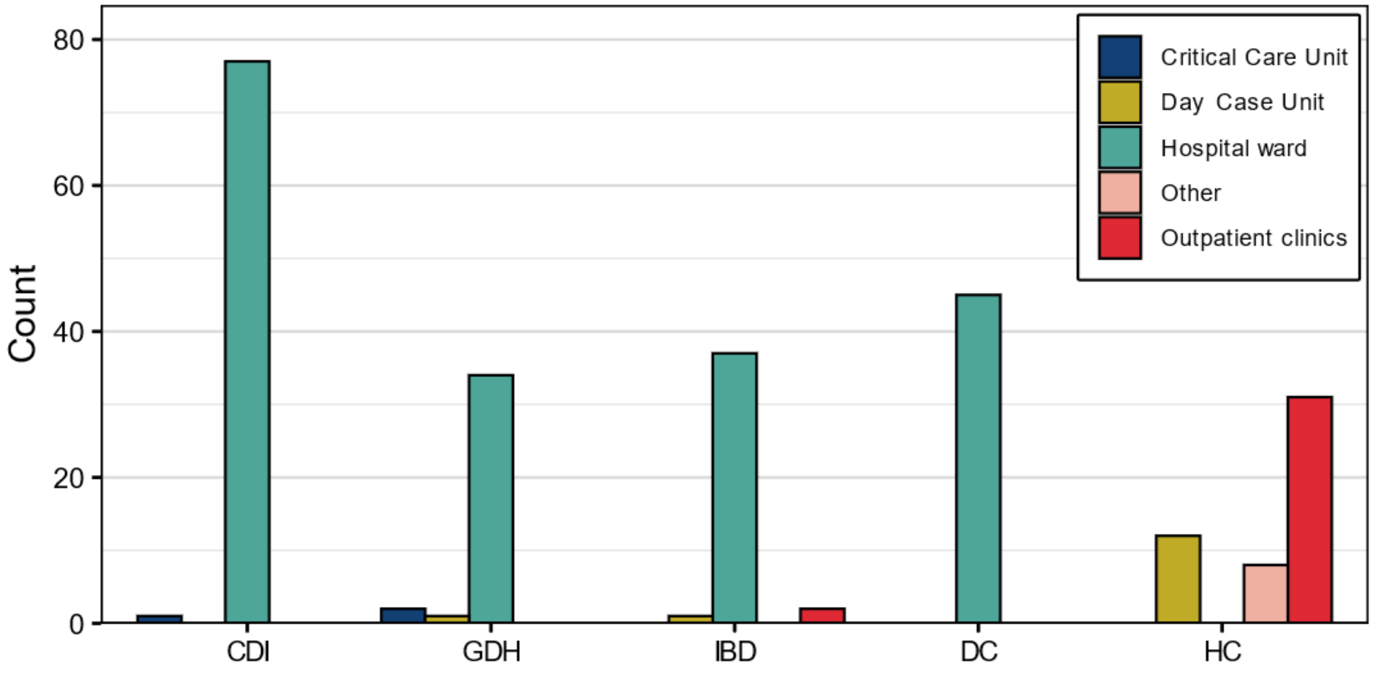

Figure S3: Recruitment source for cases and controls. 'Other' comprises patient companion and local database, 'Outpatient clinics' include hypertension, clinical biochemistry, general internal medicine, gastro-intestinal and respiratory clinics. *CDI: toxigenic C. difficile infection, GDH: non-toxigenic C. difficile infection, IBD: inflammatory bowel disease, DC: diarrhoea controls, HC: healthy controls.*

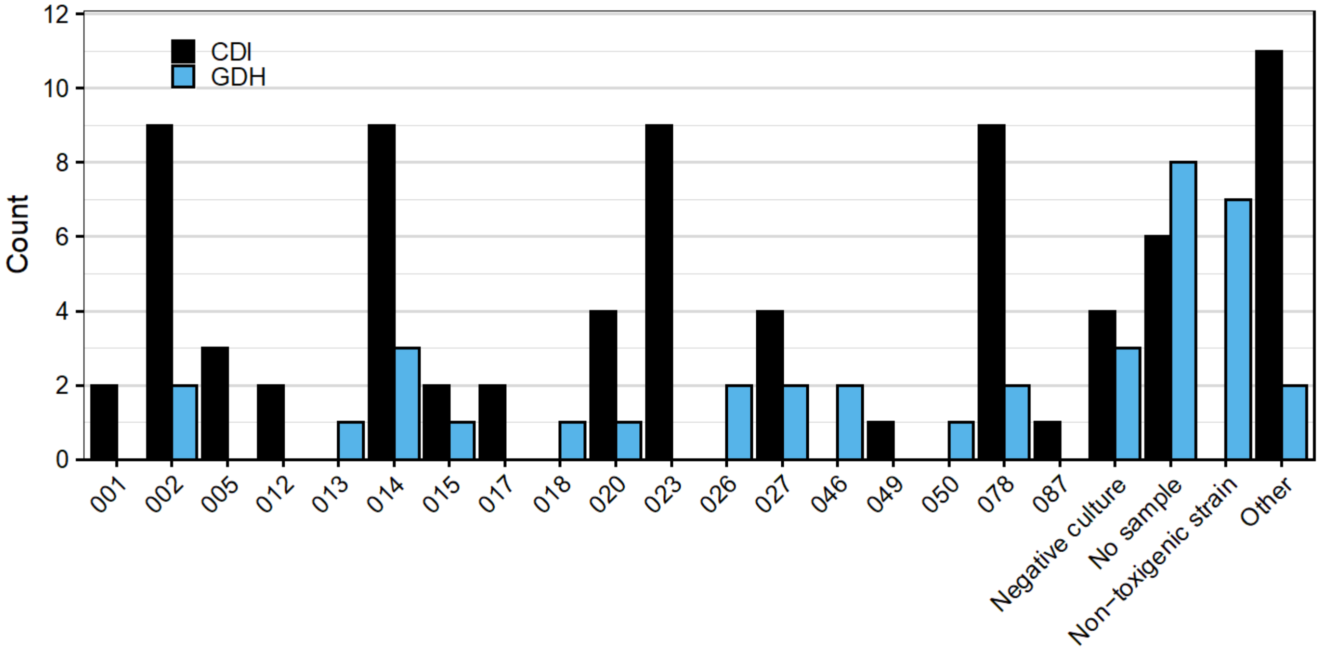

(a)

TPI	Toxin A	Toxin B	n (%)
+	+	+	11 (61)
+	-	-	7 (39)

(b)

Figure S4: Ribotypes and toxin gene presence in stools of patients with C. *difficile* infection. (**a**) Ribotypes of CDI (black) and GDH (blue) following stool culture of research and/or clinical sample, depending on availability. (**b**) Detection of toxin genes with multiplex PCR in available GDH samples (n=18, 49%). *CDI: toxigenic C. difficile infection, GDH: non-toxigenic C. difficile infection, TPI: triose-phosphate isomerase.*

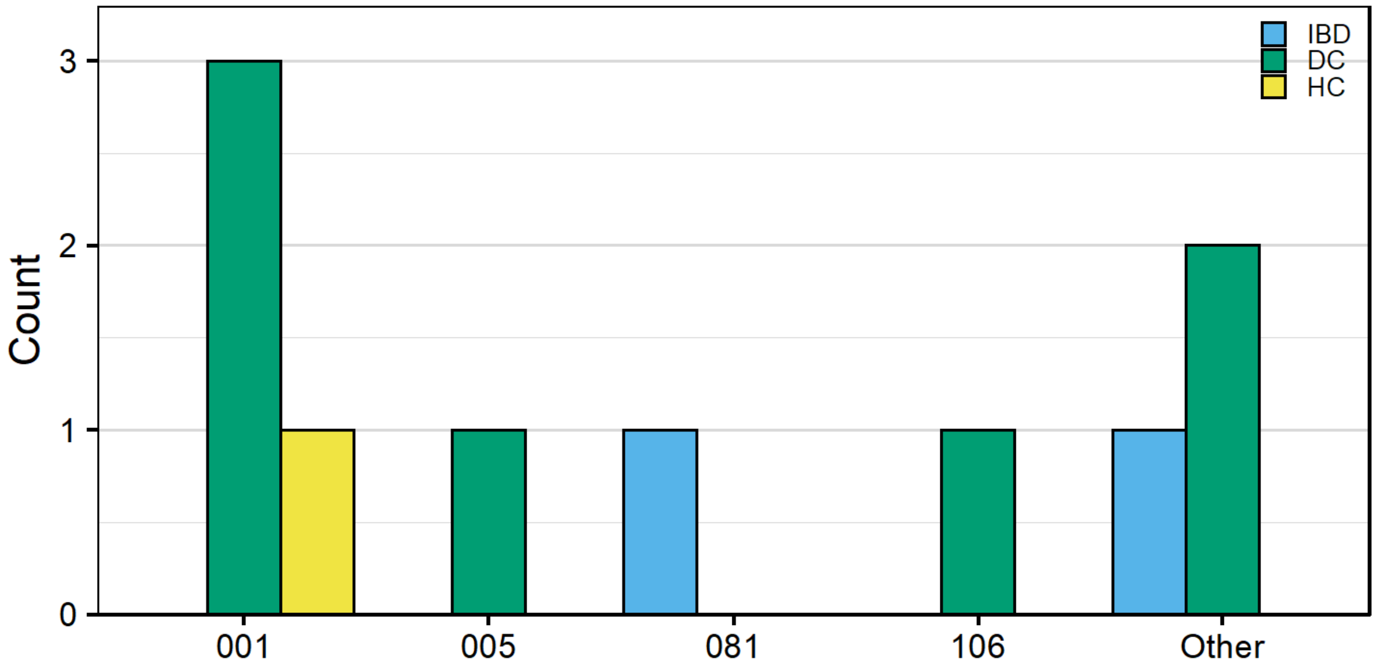

Figure S5: Positive stool cultures for *C. difficile* in control groups with GDH-/CDT-. These subjects probably represent asymptomatic *C. difficile* carriers. *GDH: glutamate dehydrogenase, CDT: C. difficile toxin, DC: diarrhoea controls, inflammatory bowel disease, HC: healthy controls*.

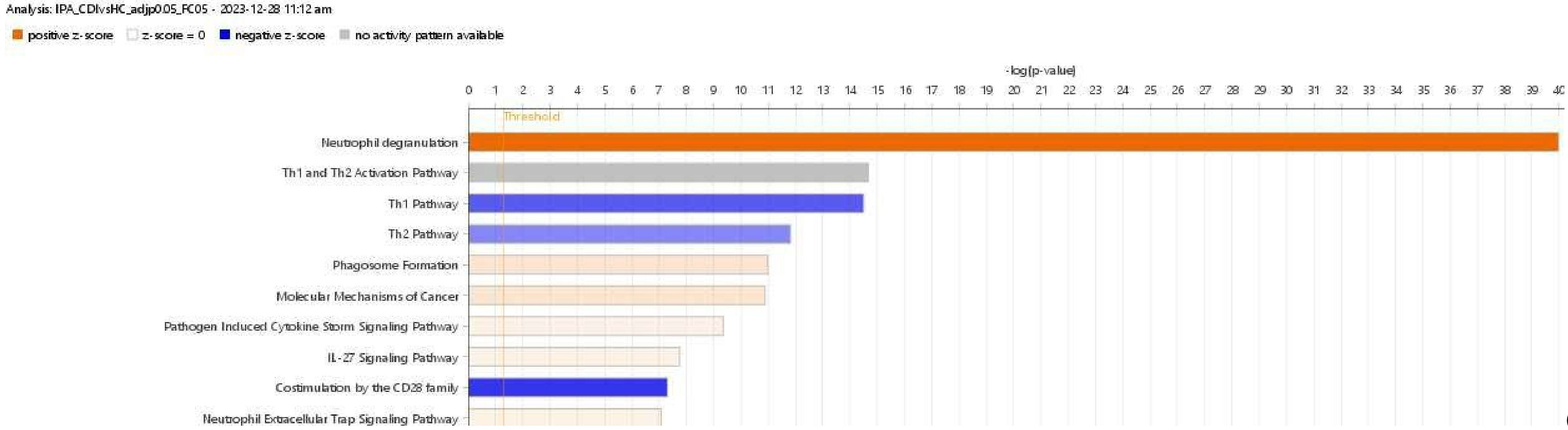

Analysis: IPA_CDIvsHC_adjp0.05_FC05 - 2023-12-28 11:12 am
positive z-score
z-score = 0
negative z-score
no activity pattern available
-log(p-value)
0 1 2 3 4 5 6 7 8 9 10 11 12 13 14 15 16 17 18 19 20 21 22 23 24 25 26 27 28 29 30 31 32 33 34 35 36 37 38 39 40
Threshold
Neutrophil degranulation
Th1 and Th2 Activation Pathway
Th1 Pathway
Th2 Pathway
Phagosome Formation
Molecular Mechanisms of Cancer
Pathogen Induced Cytokine Storm Signaling Pathway
IL-27 Signaling Pathway
Costimulation by the CD28 family
Neutrophil Extracellular Trap Signaling Pathway

(a)

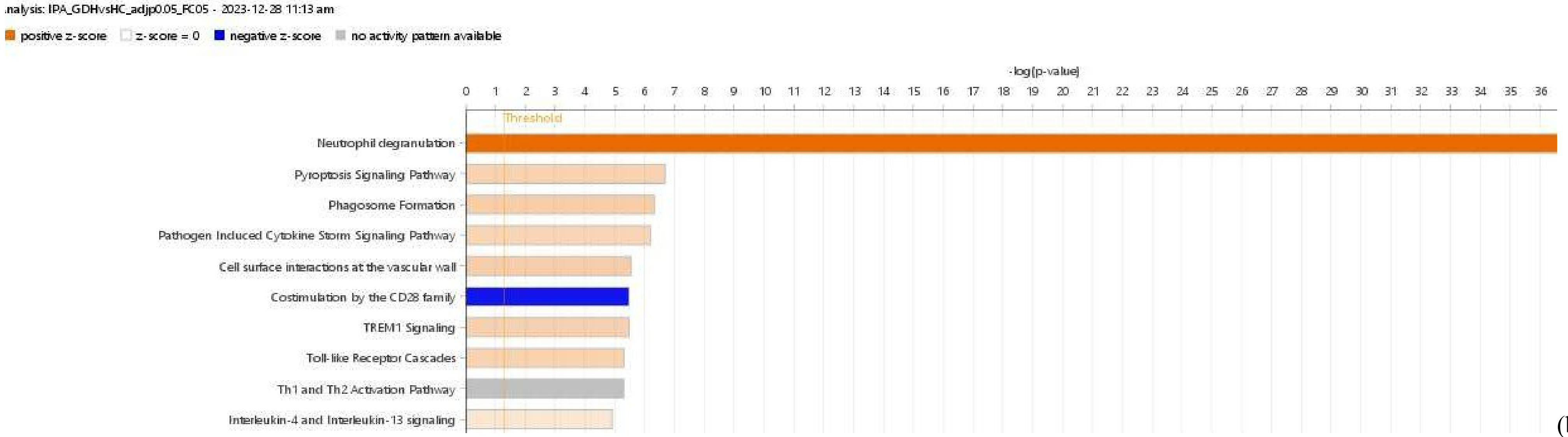

.nalysis: IPA_GDHvsHC_adjp0.05_FC05 - 2023-12-28 11:13 am
positive z-score
z-score = 0
negative z-score
no activity pattern available
-log(p-value)
0 1 2 3 4 5 6 7 8 9 10 11 12 13 14 15 16 17 18 19 20 21 22 23 24 25 26 27 28 29 30 31 32 33 34 35 36
Threshold
Neutrophil degranulation
Pyroptosis Signaling Pathway
Phagosome Formation
Pathogen Induced Cytokine Storm Signaling Pathway
Cell surface interactions at the vascular wall
Costimulation by the CD28 family
TREM1 Signaling
Toll-like Receptor Cascades
Th1 and Th2 Activation Pathway
Interleukin-4 and Interleukin-13 signaling

(b)

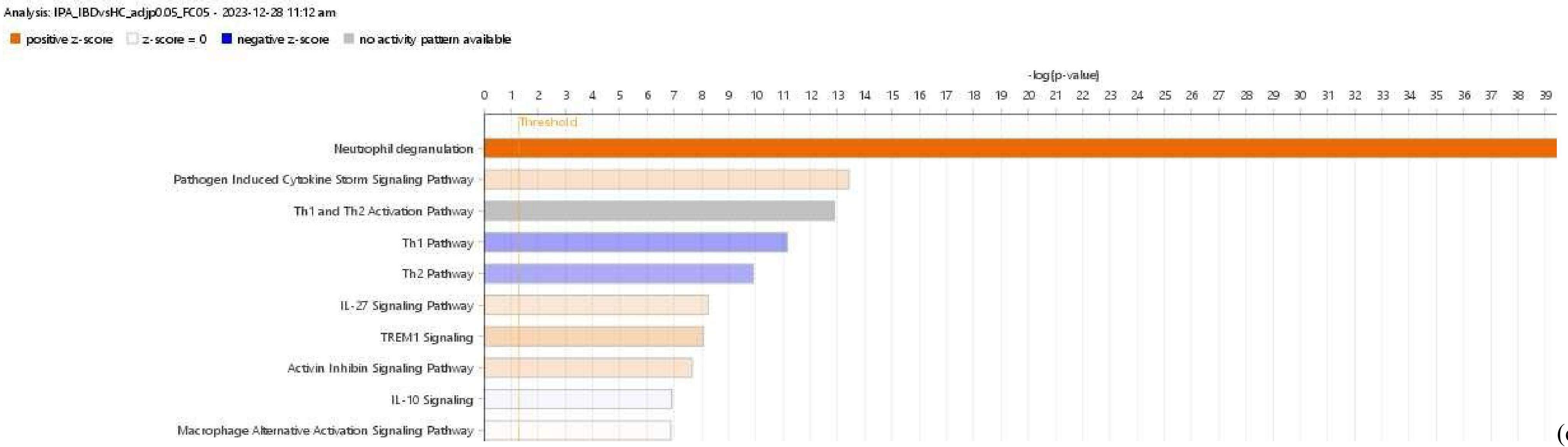

Analysis: IPA_IBDvsHC_adjp0.05_FC05 - 2023-12-28 11:12 am
positive z-score
z-score = 0
negative z-score
no activity pattern available
-log(p-value)
0 1 2 3 4 5 6 7 8 9 10 11 12 13 14 15 16 17 18 19 20 21 22 23 24 25 26 27 28 29 30 31 32 33 34 35 36 37 38 39
Threshold
Neutrophil degranulation
Pathogen Induced Cytokine Storm Signaling Pathway
Th1 and Th2 Activation Pathway
Th1 Pathway
Th2 Pathway
IL-27 Signaling Pathway
TREM1 Signaling
Activin Inhibin Signaling Pathway
IL-10 Signaling
Macrophage Alternative Activation Signaling Pathway

(c)

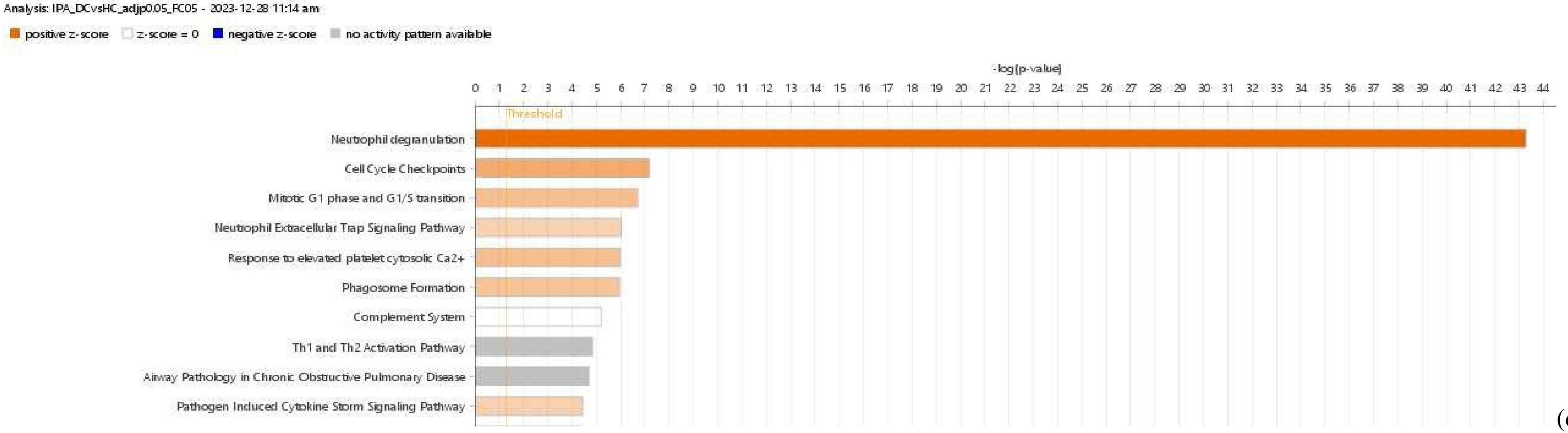

Analysis: IPA_DCvsHC_adjp0.05_FC05 - 2023-12-28 11:14 am
positive z-score
z-score = 0
negative z-score
no activity pattern available
-log(p-value)
0 1 2 3 4 5 6 7 8 9 10 11 12 13 14 15 16 17 18 19 20 21 22 23 24 25 26 27 28 29 30 31 32 33 34 35 36 37 38 39 40 41 42 43 44
Threshold
Neutrophil degranulation
Cell Cycle Checkpoints
Mitotic G1 phase and G1/S transition
Neutrophil Extracellular Trap Signaling Pathway
Response to elevated platelet cytosolic Ca2+
Phagosome Formation
Complement System
Th1 and Th2 Activation Pathway
Airway Pathology in Chronic Obstructive Pulmonary Disease
Pathogen Induced Cytokine Storm Signaling Pathway

(d)

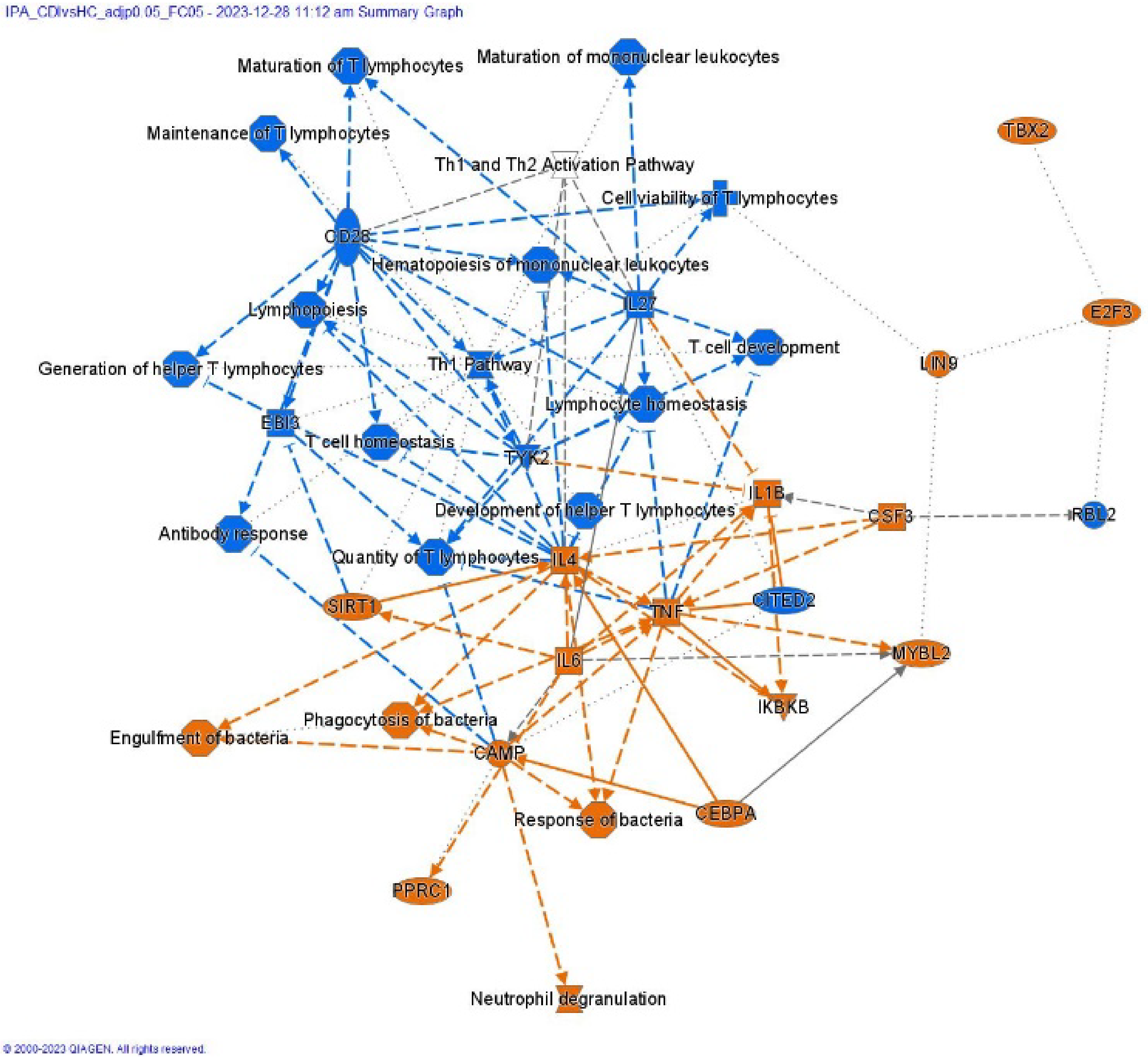
IPA_CDIvsHC_adjp0.05_FC05 - 2023-12-28 11:12 am Summary Graph
Maturation of T lymphocytes
Maturation of mononuclear leukocytes
Maintenance of T lymphocytes
Th1 and Th2 Activation Pathway
Cell viability of T lymphocytes
CD28
Hematopoiesis of mononuclear leukocytes
IL27
Lymphopoiesis
T cell development
Generation of helper T lymphocytes
Th1 Pathway
Lymphocyte homeostasis
EBI3
T cell homeostasis
TYK2
IL1B
CSF3
RBL2
Development of helper T lymphocytes
Antibody response
Quantity of T lymphocytes
IL4
SIRT1
TNF
CITED2
IL6
MYBL2
IKBKB
Phagocytosis of bacteria
Engulfment of bacteria
CAMP
Response of bacteria
CEBPA
PPRC1
Neutrophil degranulation
TBX2
E2F3
LIN9
© 2000-2023 QIAGEN. All rights reserved.

(e)

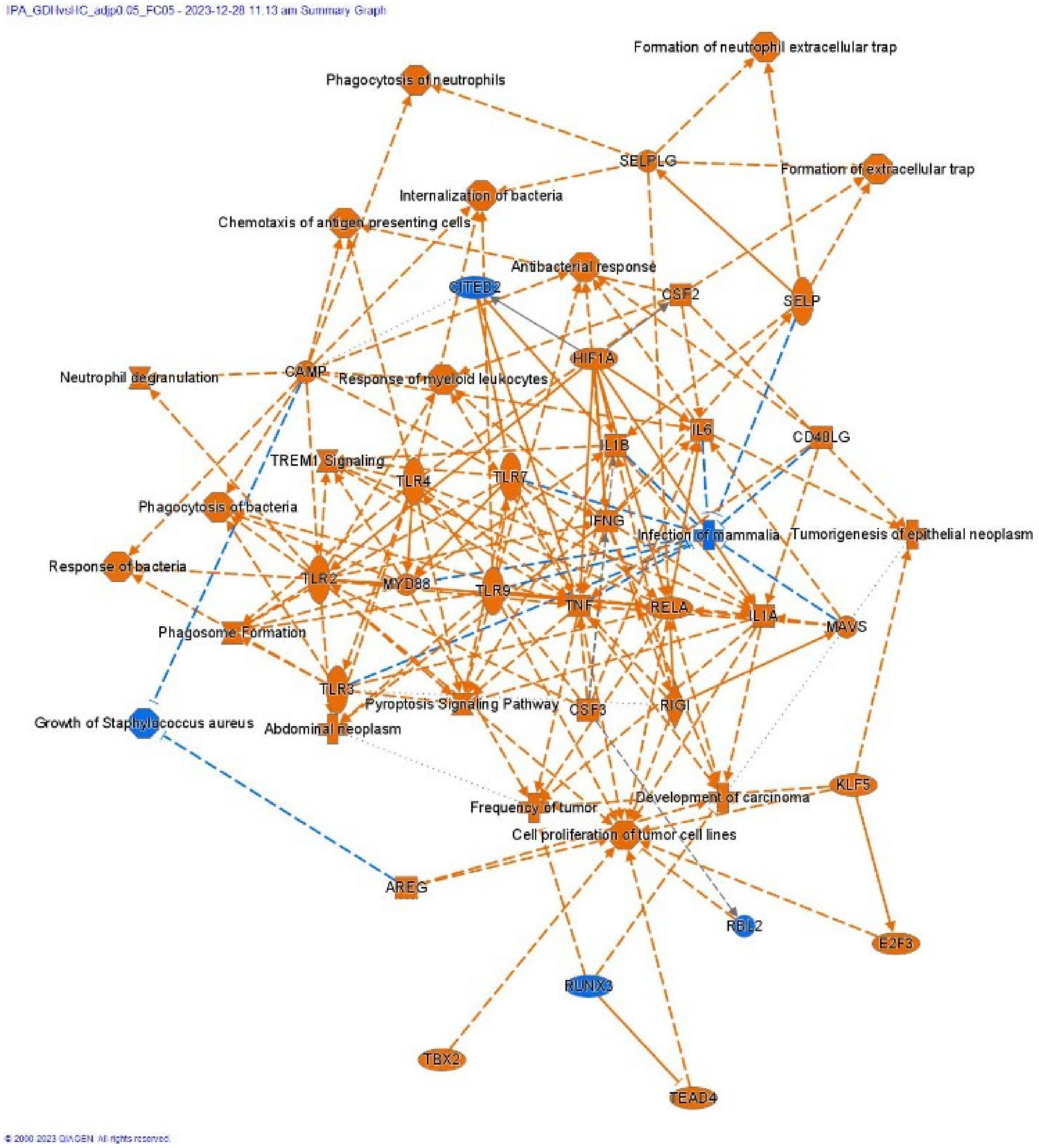
IPA_GDHvsHC_adjp0.05_FC05 - 2023-12-28 11.13 am Summary Graph
Formation of neutrophil extracellular trap
Phagocytosis of neutrophils
SELPLG
Formation of extracellular trap
Internalization of bacteria
Chemotaxis of antigen presenting cells
Antibacterial response
CITED2
CSF2
SELP
HIF1A
Neutrophil degranulation
CAMP
Response of myeloid leukocytes
IL6
CD40LG
IL1B
TREM1 Signaling
TLR4
TLR7
Phagocytosis of bacteria
IFNG
Infection of mammalia
Tumorigenesis of epithelial neoplasm
Response of bacteria
TLR2
MYD88
TLR9
TNF
RELA
IL1A
MAVS
Phagosome Formation
TLR3
Pyroptosis Signaling Pathway
CSF3
RIGI
Growth of Staphylococcus aureus
Abdominal neoplasm
KLF5
Development of carcinoma
Frequency of tumor
Cell proliferation of tumor cell lines
AREG
RBL2
E2F3
RUNX3
TBX2
TEAD4
© 2000-2023 QIAGEN. All rights reserved.

(f)

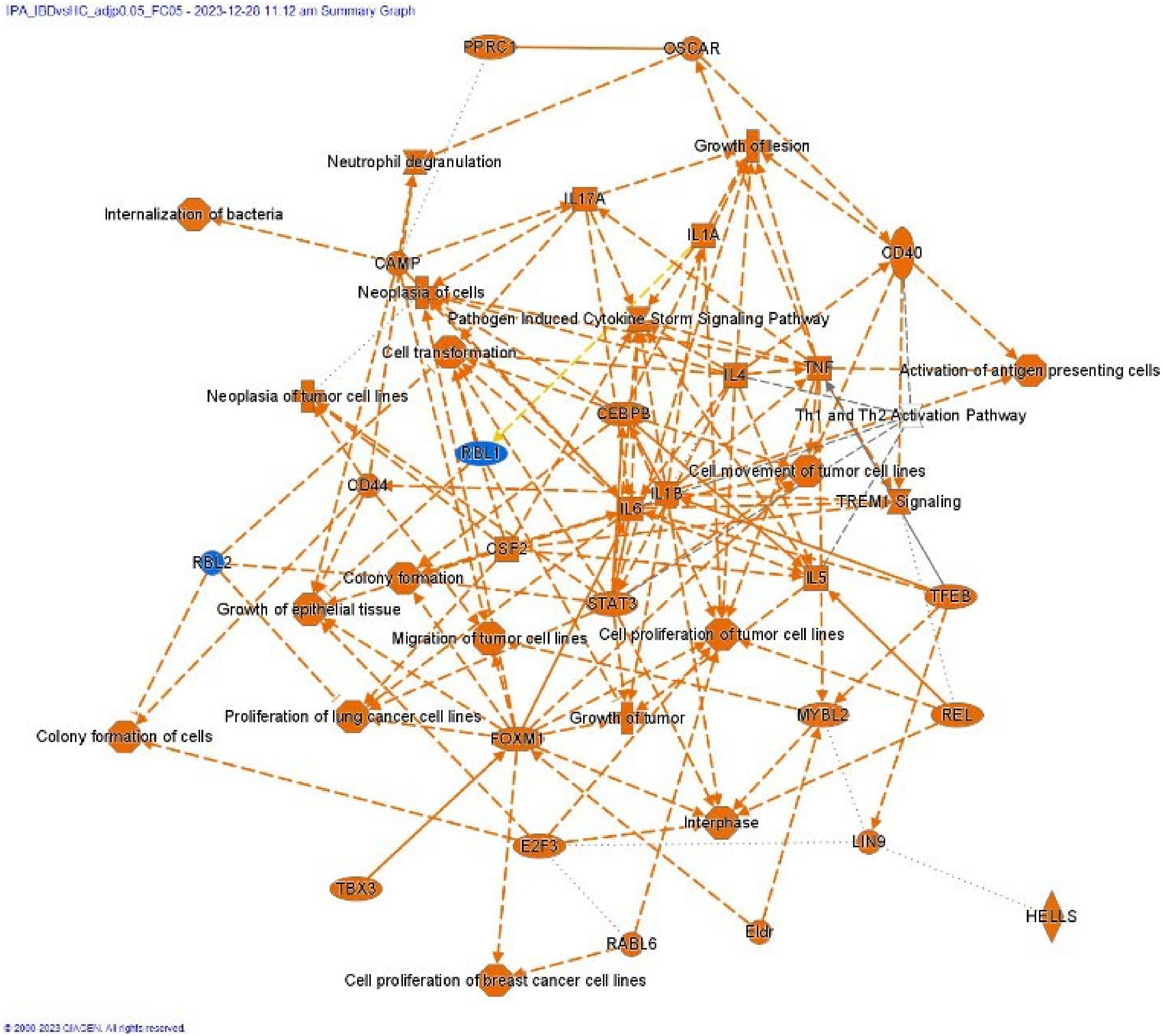

IPA_IBDvsHC_adjp0.05_FC05 - 2023-12-20 11:12 am Summary Graph
PPRC1
OSCAR
Growth of lesion
Neutrophil degranulation
Internalization of bacteria
IL17A
IL1A
CD40
CAMP
Neoplasia of cells
Pathogen Induced Cytokine Storm Signaling Pathway
Cell transformation
IL4
TNF
Activation of antigen presenting cells
Neoplasia of tumor cell lines
CEBPB
Th1 and Th2 Activation Pathway
RBL1
Cell movement of tumor cell lines
CD44
IL1B
TREM1 Signaling
IL6
CSF2
RBL2
Colony formation
IL5
TFEB
Growth of epithelial tissue
STAT3
Migration of tumor cell lines
Cell proliferation of tumor cell lines
Proliferation of lung cancer cell lines
Growth of tumor
MYBL2
REL
Colony formation of cells
FOXM1
Interphase
LIN9
E2F3
TBX3
HELLS
Eldr
RABL6
Cell proliferation of breast cancer cell lines
© 2000-2023 QIAGEN. All rights reserved.

(g)

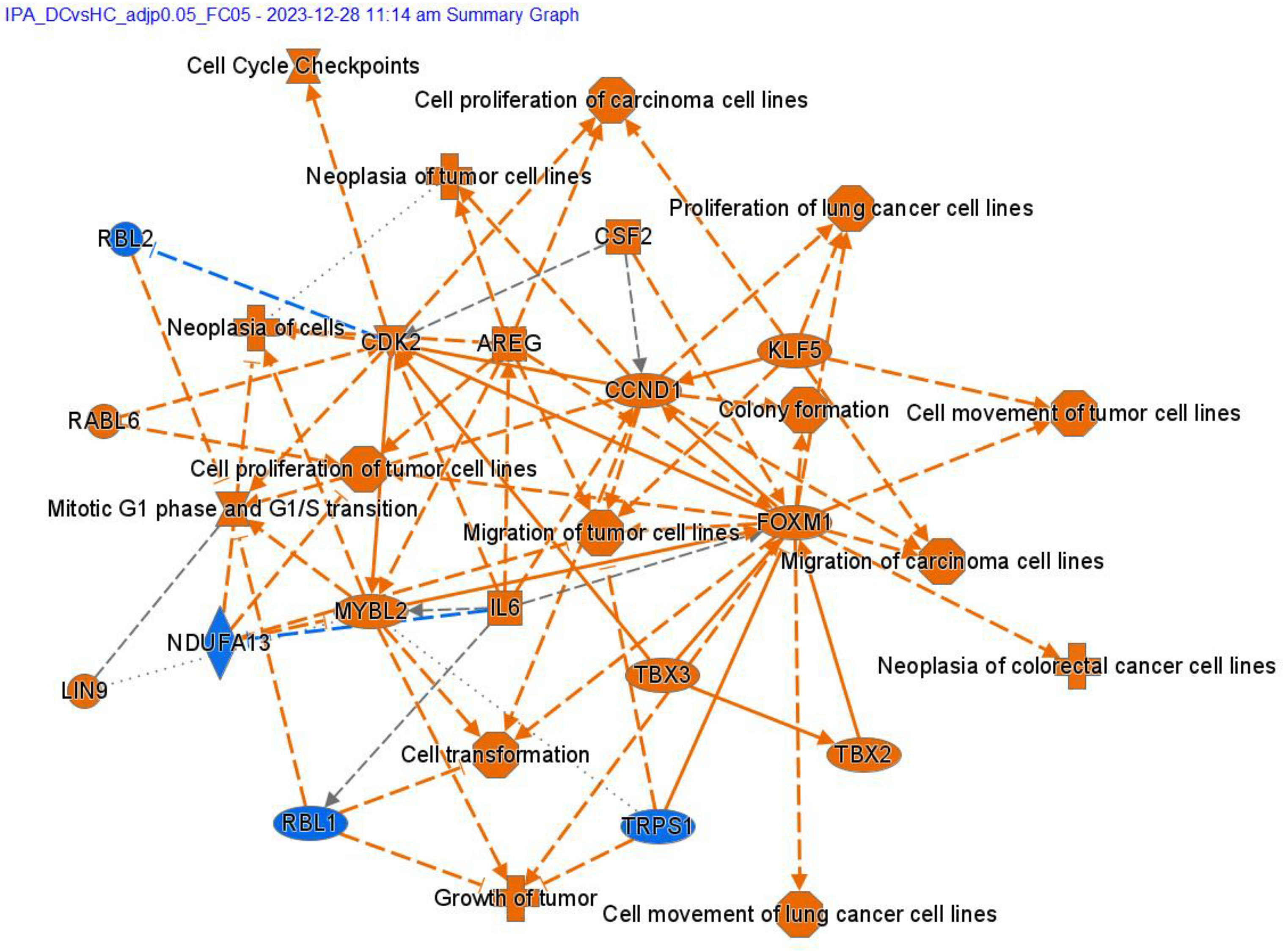

© 2000-2023 QIAGEN. All rights reserved.

(h)

Figure S6: Top 10 pathways (**a**–**d**) and summary graphs (**e**–**h**) of DE genes of diarrhoea groups vs. HC (IPA analysis; FDR adj. p <0.05 and $|\log_2 FC| > 0.5$). Neutrophil degranulation remains the most significantly upregulated (bright red) pathway in all comparisons and adaptive immunity (Th1 and Th2) pathways appear significantly downregulated in CDI only (bright blue). Graphical summaries demonstrate that gene expression is characterised by a unique downregulation in CDI compared to the other diarrhoea groups. From top to bottom: CDI vs. HC, GDH vs. HC, IBD vs. HC and DC vs. HC. *CDI: toxigenic C. difficile infection, GDH: non-toxigenic C. difficile infection, IBD: inflammatory bowel disease, DC: diarrhoea controls, HC: healthy controls, IPA: Ingenuity Pathway Analysis by Qiagen, FDR adj. p: false discovery rate adjusted p-value,* $|\log_2 FC|$*: absolute value of logarithm with base 2 of fold change.*

Figure S7: Principal component analysis of normalised gene expression data for each sample, coloured by microarray batch.

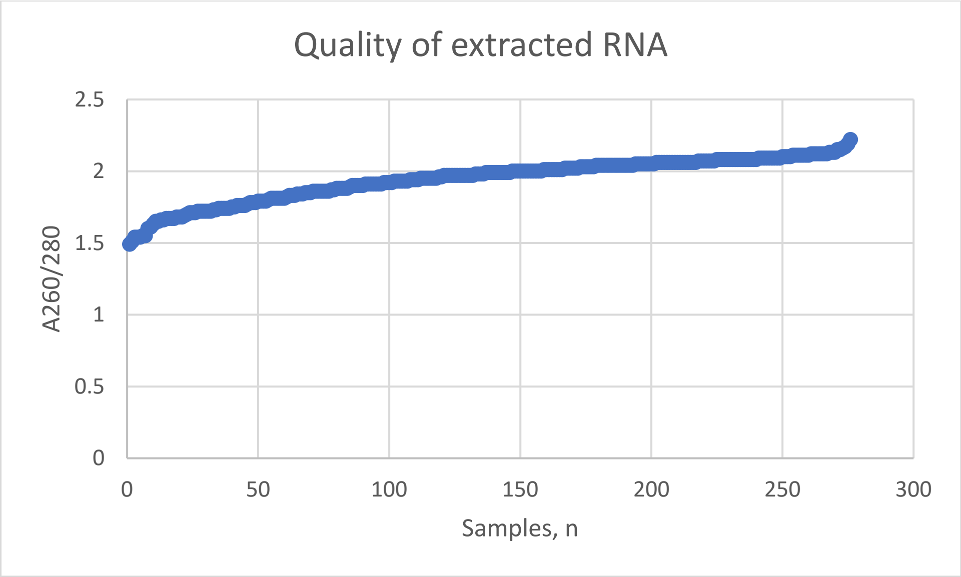
Quality of extracted RNA
A260/280
2.5
2
1.5
1
0.5
0
0
50
100
150
200
250
300
Samples, n
(a)

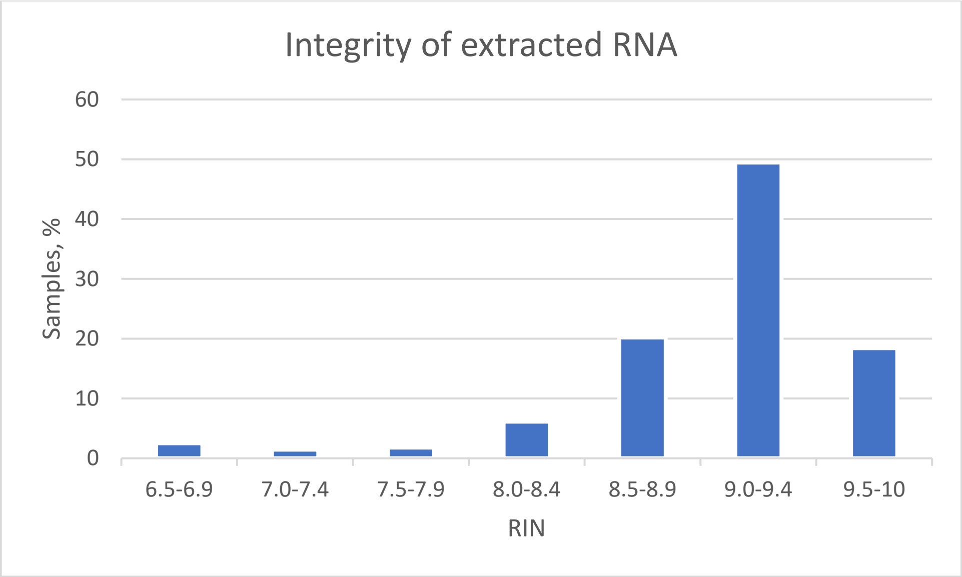
Integrity of extracted RNA
Samples, %
60
50
40
30
20
10
0
6.5-6.9
7.0-7.4
7.5-7.9
8.0-8.4
8.5-8.9
9.0-9.4
9.5-10
RIN
(b)

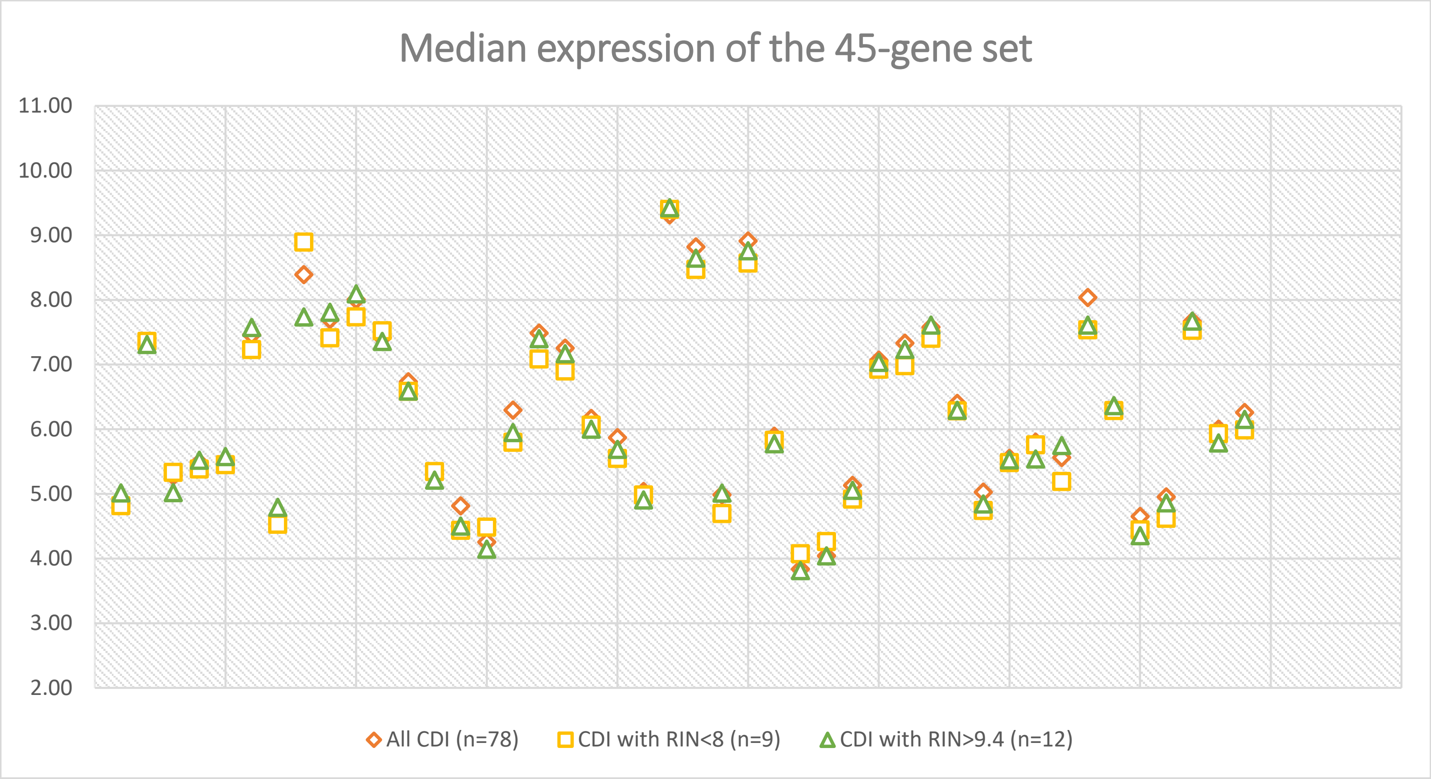
Median expression of the 45-gene set
11.00
10.00
9.00
8.00
7.00
6.00
5.00
4.00
3.00
2.00
All CDI (n=78)
CDI with RIN<8 (n=9)
CDI with RIN>9.4 (n=12)
(c)

Figure S8: Integrity and quality of extracted RNA: (a) Quality of extracted RNA was assessed with A260/280 measured with the NanoDrop Spectrophotometer (Thermo Scientific). The median A260/280 ratio of our samples was 2.0 (IQR: 1.9-2.1), which is within the range suggested by the GeneChip user guide for RNA of acceptable quality (1.7-2.1). (b) Sample stratification as per RNA integrity number RIN measured with the Agilent 2100 Bioanalyzer following RNA extraction at our laboratory. RIN measurements were confirmed at the European (Nottingham) Arabidopsis Stock Centre (NASC) and only samples with RIN ≥6.5 were processed with microarrays. The RIN threshold was satisfied by all shipped samples (data not presented). Median RIN was 9.1 (IQR: 8.8-9.4) and 94% (n=261) had RIN ≥8.0. Of the 277 samples, 251 were baseline, 25 were 2-week follow-up and 1 sample (RIN=7.6) was an outlier (very low RNA) during quality control of the arrays and was removed from the analysis. The rest 15 samples with low RIN (<8) passed the raw data inspection for outliers using relative log expression plots. (c) To investigate for differences in results between samples with low and high RIN values, we assessed gene expression in samples with RIN<8 and RIN>9.4, respectively. As most samples with low RIN (n=9, 60%) were from the CDI cohort, we focused on expression of the unique 45-gene set. The graph shows that median expression of the 45-gene set was similar for total CDI, CDI with low RIN and CDI with high RIN value. *RIN: RNA integrity number, CDI: toxigenic C. difficile infection, IQR: interquartile range.*

Table S1: Inflammatory conditions co-sharing significant gene expression patterns with our diarrhoea cohorts (C. difficile, inflammatory bowel disease, etc). Literature was investigated for members of the common 12-gene set. Top 20 upregulated genes in DE analysis (adjusted p-value <0.05) or important genes as suggested by authors were searched and overlaps with the 12-gene set are presented. This is not a systematic review of literature and the list of genes in non-exhaustive. We believe that more studies reporting common genes exist. For instance, the original publications of re-analysed datasets were not reviewed.

Condition	Overlap of diarrhoea common 12-gene set with top 20 upregulated genes (%)	Overlap of common 12-gene set with important genes (PPI, IPA or as reported by the authors)	Notes	Dataset identification number
Sepsis	*MMP8, OLFM4, CD177, CEACAM8, LTF, ANXA3, HP, ANKRD22, DEFA4* (75%)	No overlap		GSE13904, GSE54514 [183]
	OLFM4, MMP8, LTF, CEACAM8, DEFA4, CD177 (50%)	*MMP8, LTF, DEFA4, CD177*	Surgical patients with sepsis vs. surgical patients without sepsis	N/A [184]
	N/A	*LTF, DEFA4, OLFM4, CEACAM8*		GSE95233, GSE57065, GSE28750 [185]
	N/A	*CEACAM8, HP, MMP8, OLFM4, LTF, CD177*		GSE69528 [186]
	N/A	*OLFM4, CEACAM8*		GSE57065, GSE69528, GSE95233 [187]
	MMP8, CD177, OLFM4, HP, ANXA3, LTF, CEACAM8, ANKRD22 (67%)	*MMP8, CD177*	Subjects are under 18 years of age	GSE26378, GSE26440 [188]
Septic shock	From the top 9 upregulated genes: *LTF, MMP8, OLFM4* (25%)	*LTF, MMP8, OLFM4*	Septic shock vs. non-septic shock	GSE131761 [189]
	N/A	*MMP8, HP, CD177, ANKRD22, ANXA3, OLFM4, LTF, CEACAM8*	Subjects are under 18 years of age	GSE33118, GSE26440 [190]
	N/A	*CEACAM8*	Subjects are under 18 years of age	GSE26440 [191]
	OLFM4, CD177, HP, MMP8 (33%)	*CD177, OLFM4*	Survivors vs. non-survivors from septic shock	GSE132807 [192]
Septic shock (SS) and venous thromboembolism (VTE)	SS: *CD177, MMP8, HP, OLFM4, GALNT14* (42%)	Common SS and VTE: *ANXA3*		GSE64457 (SS), GSE95233 (SS), GSE57065 (SS), GSE19151 (VTE) [193]
COVID-19	N/A	*CD177, HP, GALNT14, ANXA3*		GSE171110 [194]
COVID-19, ARDS and sepsis	N/A	Common: *HP, OLFM4, DEFA4, CD177*		GSE171110 (COVID-19), GSE76293 (ARDS), GSE137342 (sepsis) [195]

H1N1	*DEFA4, HP, MMP8, LTF, CEACAM8* (42%)	*DEFA4, HP, MMP8, LTF, OLFM4*		GSE111368 [196]
Tuberculosis (Tb)/HIV-	*CEACAM8, ANKRD22, GALNT14, HP* (33%)	*CEACAM8, HP, LTF*	Subjects are under 18 years of age. Top 20 upregulated genes were common in the two datasets in active vs. latent Tb	GSE39939, GSE39940 [197]
Tb/HIV+	N/A	*DEFA4, HP, OLFM4*	Important genes were common in the two datasets in active vs. latent Tb	GSE37250, GSE69581 [198]
Osteomyelitis (OM) and VTE	N/A	Common in OM and VTE: *HP, DEFA4, LTF*		GSE30119, GSE19151, GSE48000 [199]
COPD (ITU vs. non-ICU)	N/A	*LTF, HP, CEACAM8*		E-MEXP-3589 [200]
Atherosclerotic coronary artery disease	Top 20 downregulated: *LTF, ANXA3* (17%)	*HP*		N/A [201]
Ischaemic stroke	N/A	*CEACAM8*		GSE58294, GSE16561 [202]
Haemorrhagic transformation of stroke	*OLFM4, LTF, CEACAM8* (25%)	*OLFM4, LTF, CEACAM8*	Haemorrhagic transformation vs. non-transformation of stroke following treatment	N/A [203]
Subarachnoid haemorrhage	*ANXA3, HP* (17%)	*ANXA3*		GSE36791 [204]
Ulcerative colitis	*CD177, ANKRD22, HP, SLC26A8, ANXA3, GALNT14* (50%)	*HP, CD177*		GSE94648 [50]
Systemic lupus erythematosus (SLE) associated nephritis	*LTF, MMP8, CEACAM8, DEFA4* (33%)	*LTF, MMP8, CEACAM8, DEFA4*	SLE patients with nephritis vs. SLE patients without renal involvement	GSE49454 [205]
	CEACAM8, MMP8, LTF, DEFA4, OLFM4, CD177 (50%)	*DEFA4, CD177*	Significant genes were upregulated in the blood and kidney tissue of SLE patients with nephritis.	GSE99967, GSE72798 [206]
Systemic juvenile idiopathic arthritis	*CD177, OLFM4, MMP8, CEACAM8, ANKRD22, DEFA4* (50%)	*OLFM4, MMP8, DEFA4, HP*		GSE17590, GSE80060 [207]
Juvenile dermatomyositis	*CEACAM8, DEFA4, OLFM4, LTF, MMP8* (42%)	*LTF, MMP8* (common in adult and juvenile dermatomyositis)	Top 20 upregulated gene in active adult dermatomyositis did not overlap with the top 20 upregulated in all diarrhoea common genes.	N/A [208]
Cardiovascular disease (CVD) in psoriasis	No overlap (protein-coding genes only)	*CEACAM8*	Patients with moderate to severe psoriasis with vs without CVD.	N/A [209]

Multiple sclerosis (MS)	N/A	*CD177, MMP8*	Untreated MS patients were also compared to treated MS patients with glatiramer acetate for less and more than a year and dysregulation of gene expression improves with longer treatment.	N/A [210]
Kawasaki disease	N/A	*HP, CEACAM8, MMP8, DEFA4, LTF*		GSE68004 [211]
Idiopathic pulmonary fibrosis (IPF)	Severe IPF (vs. healthy controls): *OLFM4, DEFA4, LTF, CEACAM8*<br><br>Severe vs. mild IPF: *DEFA4, OLFM4* (33%)	*OLFM4*	Mild IPF was also compared to healthy controls but there were no common genes in the top 20 upregulated with our study. Severity stratification is based on predicted DLCO; mild≥65% and severe≤35%.	GSE33566 [212]
	DEFA4, CD177, ANXA3, HP, OLFM4 (42%)	*DEFA4*		N/A [213]
Lung cancer (LC)	N/A	*ANXA3, HP* (proposed as diagnostic and prognostic markers of late-stage LC)	Controls were individuals with suspected LC but confirmed not to have LC (false positives). DE analysis was performed for all LC or non-small cell LC or late stage LC vs. false positives.	GSE198048 [214]
Primary myelofibrosis (PMF)	N/A	*DEFA4, OLFM4*		GSE26049 [215, 216]
Pre-fibrotic PMF	N/A	*MMP8, CEACAM8*		GSE26049 [217]
Longitudinal changes in pregnancy	*MMP8, CEACAM8, OLFM4, DEFA4, LTF* (42%)	*MMP8, DEFA4, LTF*	DE analysis was performed between highest vs. lowest average expression from 10-40 weeks (4-6 samples).	GSE121974 [218, 219]
Pre-eclampsia early (EP) and late (LP) onset	EP: *DEFA4*<br><br>LP: *DEFA4, CEACAM8, LTF, MMP8* (33%)	*DEFA4, LTF*	DE genes are downregulated in EP or LP	N/A [220]
Schizophrenia (SZ) and bipolar disorder (BD)	SZ: *OLFM4, CEACAM8, DEFA4, LTF, HP*<br><br>BP: *OLFM4* (42%)	*OLFM4*		N/A [221]

PPI: protein-protein interactions, IPA: ingenuity pathway analysis, N/A: not available, COVID-19: Coronavirus disease 2019, ARDS: acute respiratory distress syndrome, H1N1: Influenza A virus subtype H1N1, DE: differential expression.

Table S2: Sepsis studies presenting transcriptomic signatures with high transcriptional probability [222] and sharing genes with the common 12-gene set. Analytical approaches were mostly focused on unsupervised methods to stratify patients with gene-set classifiers. Members of the common 12-gene set were encountered in two publications which included tables of differentially expressed genes [223, 224], a multicohort analysis [225] and two classifiers [226, 227].

References	Co-shared genes (% of the 12-gene set)	Analysis	Number of DE genes (filtering criteria)
[223]	*CD177*, *MMP8* (17%)	DE between sepsis endotypes (SR1 vs. SR2) in patients with community acquired pneumonia (CAP) and top 20 upregulated genes were extracted to identify co-shared genes.	4,531 - ↑1,381 (FDR<0.05, $\lvert log_2FC \rvert \geq 0.5$)
[224]	*MMP8, CD177, ANKRD22* (25%)	DE between sepsis endotypes (SR1 vs. SR2) in patients with faecal peritonitis (FP) and top 20 upregulated genes were extracted to identify co-shared genes.	1,075 - ↑545<br><br>(FDR<0.05)
[225]	*ANKRD22, ANXA3, CD177, HP, SLC26A8* (42%)	Multicohort (n=9) GE analysis framework comparing patients with SIRS/trauma to time-matched patients with infection (leave-one-data set-out approach based on combining effect sizes and p-values).	82 (FDR <0.01, heterogeneity P >0.01, ∣summary effect size FC∣ >1.5)
[226]	33-gene set: *LTF, OLFM4* (17%)	Multi-cohort analysis identifies three clusters in sepsis patients. The four upregulated genes in the "Inflammopathic" subtype are also found in our top upregulated genes.	N/A
[227]	140-gene set: *MMP8* (8%)	MARS 1-4 endotypes bases on hierarchical consensus clustering and random forest	9,699

DE: differential expression, FDR: false discovery rate, $\lvert log_2FC \rvert$: absolute value of logarithm with base 2 of fold change, SIRS: systemic inflammatory response syndrome, FC: fold change, N/A: not available, MARS: Molecular Diagnosis and Risk of Sepsis.

Table S3: Strategy and results of GEO datasets search for colitis. The aim was to use this search to identify publications that investigate the blood transcriptome in colitis and inflammatory bowel disease to compare these findings with the common 12-gene set and differentially expressed genes in IBD, respectively. Analysis of raw data did not take place.

**Search strategy**	GEO datasets were searched for "diarrhea" OR "colitis" and "blood" in homo sapiens to identify studies investigating the blood transcriptome in colitis. Expression profiling by array and high throughput sequencing were selected in the study type filter.
**Date**	03/01/2024
**Eligibility**	Total RNA in peripheral blood was investigated in patients with diarrhoea or colitis (a). An adjusted p-value <0.05 was required for inclusion (b). Studies that used dataset from included studies were excluded (c). Publications should include a list of differentially expressed genes or other important genes (d).
**Results**	116 items -> 15 fulfilled eligibility criteria via review of title/abstract -> 7 eligible (4 studies used a non-adjusted p-value and 2 did not fulfil (d).

GEO: Gene Expression Omnibus. IBD: inflammatory bowel disease study cohort.

Table S4: Inflammatory bowel disease studies retrieved from the GEO database and investigated for members of the common 12-gene set.

References	Co-shared genes (% of the 12-gene set)	Analysis	Number of DE genes (filtering criteria)
[143]	*MMP8, OLFM4, GALNT14, CD177, MGAM2, HP, SLC26A8, ANXA3, ANKRD22* (75%)	Gene set variance analysis to establish a circulating molecular inflammation score (cirMIS) based on an intestinal MIS in IBD patients with inflamed vs. non-inflamed intestine.	N/A
[49]	*GALNT14, CD177, ANXA3* (25%)	DE analysis in patients with active IBD vs. HC with extraction of the top 20 upregulated genes.	1,627- ↑814 (adj. p <0.05)
[50]	*CD177, ANKRD22, HP, SLC26A8, ANXA3, GALNT14* (50%)	DE analysis in patients with active IBD vs. HC with extraction of the top 20 upregulated genes.	122- ↑98 (adj. p <0.05, ∣FC∣ ≥1.5
[48]	*ANXA3, OLFM4, HP* (25%)	DE analysis: CD vs. HC, UC vs. HC, CD vs. symptomatic controls (SC, including patients with C. difficile and Cambylobacter) and UC vs. SC with extraction of the top 20 upregulated genes in all comparisons. No overlap of genes for SC vs. HC and CD vs. UC.	CD vs. HC: 1,003- ↑479<br>UC vs. HC: 791- ↑421<br>SC vs. HC: 232-↑158<br>CD vs. SC: 87 -↑81<br>UC vs. SC: 65- ↑56<br>CD vs. UC: 0 (adj. p <0.05, ∣log_2FC∣> 0.5)
[228]	No overlap	DE analysis between children with IBD and controls with infectious colitis, juvenile polyp and functional intestinal disorder with extraction of the top 20 upregulated genes.	880- ↑418 (adj. p <0.05)
[229]	*OLFM4, MMP8, HP, CD177* (33%)	DE analysis in patients with severe ulcerative colitis treated with intravenous corticosteroids and responded vs. did not respond with extraction of the top 20 upregulated genes.	41 (adj. p <0.05)
[230]	*MMP8, CD177, OLFM4, HP, DEFA4, LTF, CEACAM8, ANXA3, GALNT14* (75%)	DE analysis between IBD and oligoarticular and polyarticular juvenile idiopathic arthritis (JIA) with extraction of the top 20 upregulated genes. There was no gene overlap with IBD vs systemic JIA.	IBD vs oligoarticular JIA: 1080- ↑729<br>IBD vs polyarticular JIA: 334- ↑219<br>IBD vs systemic JIA: 90- ↑22<br>(adj. p <0.05, ∣log_2FC∣ >0.5)

DE: differential expression, IBD: inflammatory bowel disease, N/A: not available, HC: healthy controls, adj. p: adjusted p-value, ∣FC∣: absolute value of fold change, CD: Chron's disease, UC: ulcerative colitis, ∣log_2FC∣: absolute value of logarithm with base 2 of fold change.

Table S5: Criteria for severe *C. difficile* infection as per the UK Health Security Agency [46] and the European Society of Clinical Microbiology and Infectious Diseases [47] and modified criteria to apply to all patients with diarrhoea in this study.

Severe *C. difficile* infection	Severe diarrhoea disease (modified criteria)
WCC >15x10^9 /L, or	WCC >15x10^9 /L, or
Serum creatinine >50% increase above baseline, or	Serum creatinine >133 µmol/L, or
Temperature >38.5°C, or	Temperature >38.5°C
Evidence of severe colitis (abdominal or radiological signs)	

WCC: white cell count.

Table S6: Expression changes (log_2FC and FDR adj. p) of the common 12-gene set in diarrhoea groups vs. HC, CDI repeat sampling and severe diarrhoea. All genes were downregulated in two weeks following infection and the decrease was statistically significant in most cases. The expression of the common 12-gene set was higher in severe vs. non-severe cases but the difference was less than 1 log_2 and not always statistically significant.

**Diarrhoea common 12-gene set**	**CDI vs. HC**		**GDH vs. HC**		**IBD vs. HC**		**DC vs. HC**		**Baseline vs. 2-week follow-up (n=25 CDI cases)**		**Severe diarrhoea vs. non-severe diarrhoea**	
	Log_2FC	FDR adj. p-value	Log_2FC	FDR adj. p-value	Log_2FC	FDR adj. p-value	Log_2FC	FDR adj. p-value	Log_2FC	FDR adj. p-value	Log_2FC	FDR adj. p-value
ANKRD22	1.814056835	2.59E-11	1.659017439	6.37E-07	2.32562273	1.68E-11	1.648628025	9.26E-08	0.18704	0.57096	0.45042	0.027041
ANXA3	1.892008142	1.01E-16	1.374370654	3.55E-07	2.678022656	1.12E-19	1.50163047	2.63E-09	0.696532	0.03369	0.44377	0.015041
CD177	2.243485457	9.05E-11	1.441317558	0.000817504	3.000294731	1.02E-11	1.969273057	5.64E-07	1.183322	0.012826	0.77791	0.015041
CEACAM8	2.551248048	1.96E-14	1.874700615	2.76E-06	3.089129722	1.69E-13	1.473972847	7.13E-05	1.154299	0.027123	0.37447	0.120316
DEFA4	2.287590158	2.55E-11	1.876078098	7.89E-06	2.934131334	1.62E-11	1.34809815	0.0006262	1.233077	0.022728	0.36008	0.135955
GALNT14	2.454339312	1.28E-19	1.886912261	3.73E-09	3.06820147	2.26E-19	1.999603241	1.98E-11	1.115396	0.010645	0.51993	0.015041
HP	1.975782922	5.67E-13	1.448844	1.32E-05	2.721746545	1.02E-14	1.426202818	3.54E-06	0.933792	0.022728	0.5342	0.016044
LTF	2.481337316	2.85E-14	1.926331484	8.95E-07	3.01070536	2.28E-13	1.668263554	4.07E-06	1.244574	0.022728	0.40532	0.092662
MGAM2	2.239524328	2.44E-21	1.734854528	7.24E-10	2.327791441	6.18E-16	1.633225593	1.69E-10	0.560396	0.07205	0.45921	0.015041
MMP8	3.573576224	6.83E-17	2.760586902	5.69E-08	4.539505628	5.81E-17	2.376159482	3.53E-07	1.892294	0.012826	0.66556	0.034387
OLFM4	2.87183321	4.84E-12	1.620312967	0.001674184	4.1606532	7.95E-15	1.894384376	5.54E-05	1.88615	0.012826	0.53349	0.097353
SLC26A8	1.898886571	4.17E-17	1.659782214	1.81E-09	2.722265794	1.80E-20	1.572211809	3.49E-10	0.593048	0.03993	0.38229	0.027041

CDI: toxigenic C. difficile infection, HC: healthy controls, GDH: non-toxigenic C. difficile infection, IBD: inflammatory bowel disease, DC: diarrhoea controls, log_2FC: logarithm with base 2 of fold change, FDR adj. p: false discovery rate adjusted p-value.
